# Effects of chronic noise exposure on the microbiome-gut-brain axis in senescence-accelerated prone mice: implications for Alzheimer’s disease

**DOI:** 10.1186/s12974-018-1223-4

**Published:** 2018-06-22

**Authors:** Bo Cui, Donghong Su, Wenlong Li, Xiaojun She, Ming Zhang, Rui Wang, Qingfeng Zhai

**Affiliations:** 1 Department of Operational Medicine, Tianjin Institute of Environmental and Operational Medicine, Tianjin, China; 2 School of Medicine and Life Sciences, University of Jinan-Shandong Academy of Medical Sciences, Jinan, China; 3 Shandong Academy of Occupational Health and Occupational Medicine, Jinan, China; 4 School of Public Health and Management, Weifang Medical University, Weifang, China; 5 Tianjin Centers for Disease Control and Prevention, Tianjin, China

**Keywords:** Noise, Alzheimer’s disease (AD), Microbiome-gut-brain axis, Inflammation, SAMP8 mouse

## Abstract

**Background:**

Chronic noise exposure is associated with neuroinflammation and gut microbiota dysregulation and increases the risk of Alzheimer’s disease (AD). Environmental hazards are also thought to be associated with genetic susceptibility factors that increase AD pathogenesis. However, there is limited experimental evidence regarding the link between chronic noise stress and microbiome-gut-brain axis alterations, which may be closely related to AD development.

**Methods:**

The aim of the present study was to systematically investigate the effects of chronic noise exposure on the microbiome-gut-brain axis in the senescence-accelerated mouse prone 8 (SAMP8) strain. We established SAMP8 mouse models to examine the consequences of noise exposure on the microbiome-gut-brain axis. Hippocampal amyloid-β (Aβ) assessment and the Morris water maze were used to evaluate AD-like changes, 16S ribosomal RNA sequencing analyses were used for intestinal flora measurements, and assessment of endothelial tight junctions and serum neurotransmitter and inflammatory mediator levels, as well as fecal microbiota transplant, was conducted to explore the underlying pathological mechanisms.

**Results:**

Chronic noise exposure led to cognitive impairment and Aβ accumulation in young SAMP8 mice, similar to that observed in aging SAMP8 mice. Noise exposure was also associated with decreased gut microbiota diversity and compositional alterations. Axis-series studies showed that endothelial tight junction proteins were decreased in both the intestine and brain, whereas serum neurotransmitter and inflammatory mediator levels were elevated in young SAMP8 mice exposed to chronic noise, similar to the observations made in the aging group. The importance of intestinal bacteria in noise exposure-induced epithelial integrity impairment and Aβ accumulation was further confirmed through microbiota transplantation experiments. Moreover, the effects of chronic noise were generally intensity-dependent.

**Conclusion:**

Chronic noise exposure altered the gut microbiota, accelerated age-related neurochemical and inflammatory dysregulation, and facilitated AD-like changes in the brain of SAMP8 mice.

**Electronic supplementary material:**

The online version of this article (10.1186/s12974-018-1223-4) contains supplementary material, which is available to authorized users.

## Background

Alzheimer’s disease (AD) is a common age-related central nervous system (CNS) disorder that is associated with neurodegeneration and cognitive deficits. At present, the pathogenesis of AD is generally considered to involve the interaction of several internal (individual) and external environmental factors [1, 2]. Internal factors contributing to AD include genetics, aging, and other factors that are largely inherited and cannot be changed. External environmental factors affecting the risk of AD include chronic exposure to physical, chemical, and psychosocial hazards, as well as lifestyle factors.

It has been reported that the incidence of AD doubles every 5 years after the age of 65 years, emphasizing the role of aging as a principal risk factor for AD [3]. Furthermore, environmental factors, which may act to induce oxidative stress and concomitant inflammation, may play a key role in the etiology of AD [4]. For example, chronic noise exposure has been associated with AD-like cognitive dysfunction as well as persistent tau and amyloid-β (Aβ) pathology [5–9]. Thus, environmental health hazards, such as noise exposure, might be associated with an increased risk of developing AD. Moreover, it can be speculated that the combined effects of environmental noise exposure and aging may play a larger role than individual factors in the onset of AD.

Approximately 95% of symbiotic microbes are located in the gut. These microbes play major roles in human nutrition, digestion, neurotrophy, inflammation, growth, and immunity [10]. The gut-brain axis is a complex bidirectional communication network [11] in which the gut microbiota facilitates signaling communication. As a result, the concept of the gut-brain axis can be more accurately reconceived as the microbiota-gut-brain axis, a system that modulates immune, gut, and CNS functions [12]. Many nervous system dysfunctions have been associated with the differential abundance and composition of the gut microbiota [13–15]. Gut microbiota disruption influences the production of neurotransmitters in the gut, which can then affect their levels in the CNS [16]. On the other hand, noise-induced dysfunction of the GABAergic and monoaminergic systems may contribute to cognitive impairment [17, 18]. Moreover, we previously investigated the effects of noise exposure on gut microbiota and glucose metabolism and found that chronic noise exposure altered the composition of the gut microbiota and negatively affected glucose metabolism, host immune responses, and insulin activity [19].

While the effects of chronic environmental noise exposure on brain function, AD-like pathology, and the gut microbiota are unclear, we speculate that there might be cooperative interactions between noise exposure and aging that increase the risk of AD, and that the microbiome-gut-brain axis plays an important role in this process. To examine this hypothesis, we selected the senescence-accelerated mouse prone 8 (SAMP8) strain, a spontaneous mouse model of accelerated aging, and investigated the effects of chronic noise exposure on cognitive performance, hippocampal Aβ deposition, gut microbiota composition, and serum neurotransmitters and inflammatory mediators.

## Methods

### Animals and experimental groups

The Tianjin University of Traditional Chinese Medicine kindly provided the SAMP8 mice. Mice were maintained in the laboratory animal center of the Tianjin Institute of Environmental and Operational Medicine under standard housing conditions (ambient temperature 23 ± 2 °C and 50–60% humidity on a 12-h light/dark cycle (lights on at 06:00)). Animals were allowed to adapt to the laboratory environment for 4 days before the start of the experiment. All mice were provided with a standard laboratory rodent diet (Laboratory Animal Center, Tianjin Institute of Environmental and Operational Medicine) and water ad libitum. Three-month-old male SAMP8 mice were randomly separated into control, low-intensity noise exposure (LN), and high-intensity noise exposure (HN) groups. Eight-month-old male SAMP8 mice were used as positive controls (aging group). Ten mice were used per group. The LN and HN groups were exposed to 88 or 98 dB sound pressure level (SPL) white noise (4 h/day for 30 days from 8:00 to 12:00), respectively, whereas control mice were housed in similar cages and exposed to background noise (< 40 dB SPL) from another chamber. After noise exposure for 30 consecutive days, the animals were individually subjected to Morris water maze testing and subsequently sacrificed for the collection of blood by cardiac puncture and brain, intestine, and cecal content sampling. All samples were stored at − 80 °C until use. The Institutional Animal Use and Care Committee of the Tianjin Institute of Environmental and Operational Medicine approved the animal treatment, husbandry, and experimental protocols used in this study.

### Noise exposure setup

Noise exposure was provided as described previously [8]. Briefly, noise was generated using a noise generator (BK 3560C, B&K Instruments, Denmark), amplified with a power amplifier (Yong-Sheng Audio P-150D; The Third Institute of China Electronic Technology Group, Beijing, China), and delivered through a loudspeaker (ZM-16S; Tianjin Zenmay Electroacoustic Equipment Co., Tianjin, China). The main spectrum of the noise signal from the generator was in the range of 20–20,000 Hz. Animals were exposed to noise in a reverberation chamber in wire mesh cages placed at the center of the sound field. A loudspeaker was suspended directly above the cages.

### Morris water maze testing

Morris water maze testing was performed in accordance with our previous study [17]. The behavioral test included a hidden platform training (spatial learning) and a probe trial (spatial memory) session. In the first session, mice searched for a hidden platform located 2 cm below the surface of the water. Mice were placed into the water facing the pool wall in one of the four quadrants (the location alternated in a clockwise manner for each trial). When the mouse located the platform, it was allowed to stay on the platform for 10 s before the trial was ended. If the mouse failed to locate the platform within 60 s, it was manually placed on the platform and left there for 10 s. Mice completed four trials per day for four consecutive days. In the probe trial session on day 5, the platform was removed and each mouse was allowed to swim freely for 60 s. During behavioral testing, animal movements were recorded with a video camera and were analyzed using Animal Behavior Video Analysis System (v.3.1; Shanghai XinRun Information Technology Co. Ltd., Shanghai, China) for quantification of the latency to reach the hidden platform, time spent in the target quadrant during the training sessions, time spent in the target quadrant during the probe trial, and the number of times that mice crossed the location of the previous trial’s platform in the probe trial.

### Enzyme-linked immunosorbent assay

Intracardiac blood was drawn, and serum was obtained by centrifuging for 10 min at 3000*g*. The serum was then stored at − 80 °C until the assays were performed. Blood serum levels of 5-hydroxytryptamine (5-HT), GABA, endotoxin (ET), and corticosterone were measured using mouse enzyme-linked immunosorbent assay (ELISA) kits (BlueGene Biotech, Shanghai, China) according to the manufacturer’s instructions. The mean value of duplicate samples was taken as the final concentration for each animal.

### Determination of gene expression by real-time polymerase chain reaction

Colon tissues were removed from the mice following the last session of noise exposure and homogenized using a rapidly oscillating masher. Total RNA was extracted using the RNeasy Mini kit (TaKaRa Bio, Dalian, China) in accordance with the manufacturer’s instructions. Total RNA was converted to complementary DNA (cDNA) by reverse transcription using the Transcriptor First Strand cDNA Synthesis kit (TaKaRa Bio, Dalian, China). A primer pair designed to amplify the β-actin gene was used as an internal control. The specific primers and probes designed for mouse claudin (CLDN)1, CLDN2, CLDN3, CLDN8, CLDN15, occludin, tight junction protein 1 (ZO-1), tight junction protein 2 (ZO-2), and β-actin are described in Table 1. Gene expression levels were assessed by quantitative real-time polymerase chain reaction (PCR) performed under the following thermal cycling conditions: 2 min at 50 °C and 10 min at 95 °C followed by 45 cycles of 95 °C for 5 s and 57 °C for 30 s. Real-time PCR was performed using gene expression assays on demand and a Takara PCR Thermal Cycler Dice Real-Time system (TaKaRa Bio, Dalian, China). The threshold cycle (Ct) of target genes was normalized to that of β-actin. Messenger RNA (mRNA) levels in noise-exposed animals were calculated after normalizing cycle thresholds to β-actin expression and are presented as fold induction values (2^−ΔΔCt^) relative to control mice.

**Table 1:** Primers for the detection of mouse gene products by quantitative real-time PCR

Gene	Primers
Occludin	F: 5′-CAGGTGAATGGGTCACCGAG-3′
	R: 5′-TCAAAAGGCCTCACGGACAT-3′
ZO-1	F: 5′-GAACTTTGACCTCTGCAGCAA-3′
	R: 5′-AGAAATCGTGCTGATGTGCC-3′
ZO-2	F: 5′-CAGCTTGTAGTTCTGAGCCG-3′
	R: 5′-ATGACGATTGACGTCTCCCC-3′
CLDN1	F: 5′-CCACCATTGGCATGAAGTGC-3′
	R: 5′-CTGGCATTGATGGGGGTCAA-3′
CLDN2	F: 5′-ATGCCTTCTTGAGCCTGCTT-3′
	R: 5′-AAGGCCTAGGATGTAGCCCA-3′
CLDN3	F: 5′-GCAAGGACTACGTCTGAGGG-3′
	R: 5′-ACTGTGTGTCGTCTGTCACC-3′
CLDN8	F: 5′-GGAATGCCAATCCATCACGC-3′
	R: 5′-AGGCGTGTAGAGGGAAAGGA-3′
CLDN15	F: 5′-TCCGTGACATCCCTTTTGGG-3′
	R: 5′-TTGCCATGGACCGTAGACAC-3′
β-Actin	F: 5′-CTACAATGAGCTGCGTGTGG-3′
	R: 5′-AAGGAAGGCTGGAAGAGTGC-3′

*ZO-1* tight junction protein 1, *CLDN* claudin

### Assessment of inflammatory factors by protein array

The 40-cytokine preconfigured RayBio^®^ Mouse Inflammation Antibody Array kit (GSM-INF-1-1; RayBiotech, Norcross, GA, USA) was used to quantify inflammatory mediators in blood serum samples. The chip was read using a GenePix 4000B Microarray Scanner (Molecular Devices, Sunnyvale, CA, USA). The array was arranged in such a manner that each antibody was spotted fourth, creating four replicates per protein of interest. Specific protocol details can be found at the RayBiotech, Inc., website (http://www.raybiotech.com). The layout of the spotted primary antibodies is shown in Table 2.

**Table 2:** Primary antibody map showing the antibody layout for each array on the microarray chip (RayBio^®^ G-Series Mouse Inflammation Array 1; image adapted from RayBiotech)

	Each antibody is printed in quadruplicate horizontally											
	1	2	3	4	1	2	3	4	1	2	3	4
A	POS1				POS2				BLC (CXCL13)			
B	CD30 ligand				Eotaxin-1 (CCL11)				Eotaxin-2 (MPIF-2)			
C	Fas ligand				GCSF				GM-CSF			
D	ICAM-1 (CD54)				IFN-gamma				L-1 alpha			
E	IL-1beta				IL-2				IL-3			
F	lL-4				IL-5				IL-6			
G	IL-7				IL-10				IL-12 p70			
H	IL-15				IL-15				IL-17A			
I	IL-21				KC (CXCL1)				Leptin			
J	LIX				MCP-1 (CCL2)				MCP-5			
K	M-CSF				MIG (CXCL9)				MIP-1 alpha (CCL3)			
L	MIP-1 gamma				Platelet factor 4				RANTES (CCL5)			
M	TARC (CCL17)				1–309 (TCA-3)				TIMP-1			
N	TNF-alpha				TNF RI				TNF RII			

*MIP-1* macrophage inflammatory protein 1

### Western blot analysis

Hippocampi were dissected immediately after the animal was sacrificed and were stored at − 80 °C until use. For immunoblot analysis, frozen hippocampi were homogenized in ice-cold 50 mM Tris-HCl buffer (pH 7.4) containing 1% Triton X-100, 0.2 mM PMSF, and 1 mM EDTA. Homogenates were centrifuged at 12,000*g* for 10 min at 4 °C. The supernatants obtained were immediately placed in boiling water for 10 min. Samples (10 μg protein/lane) were separated on 10% SDS-PAGE gels and electrophoretically transferred to nitrocellulose membranes. Membranes were probed with rabbit antibodies against the following proteins: Aβ (1:200; Santa Cruz, USA), claudin 1 (1:1000; Bioworld, USA), claudin 3 (1:1000; Bioworld, USA), occludin (1:800; Bioworld, USA), ZO-1 (1:500; Proteintech, USA), and glyceraldehyde phosphate dehydrogenase (GAPDH) (1:10,000; Proteintech, USA); GADPH was used as an internal reference standard. Peroxidase-conjugated goat anti-rabbit IgG (H+L) (ZSGB-BIO, China) was used as a secondary antibody.

### Sequencing of the 16S ribosomal RNA gene in microbiota

16S ribosomal RNA (rRNA) gene sequencing was performed as per our previous study [19]. Briefly, DNA was extracted from cecal content samples using the cetyltrimethylammonium bromide/sodium dodecyl sulfate method. Distinct regions of 16S rRNA genes were amplified using specific primers (16S V4, 515F: GTGCCAGCMGCCGCGGTAA, 806R: GGACTACHVGGGTWTCTAAT) with a barcode. All PCR reactions were performed with Phusion^®^ High-Fidelity PCR Master Mix (New England Biolabs). The 16S rRNA gene was analyzed to evaluate bacterial diversity using Illumina HiSeq (Novogene Bioinformatics Technology Co., Ltd., Beijing, China). Sequences were analyzed using the Quantitative Insights Into Microbial Ecology software (http://qiime.org/). A representative sequence was selected for each operational taxonomic unit (OTU), and the Ribosomal Database Project classifier was used to annotate taxonomic information for each representative sequence.

### Fecal microbiota transplantation

After noise exposure for 30 consecutive days, donor fecal microbiota was collected from control SAMP8 mice and the HN group mice and transferred to the recipient mice. The recipients, male 3-month-old SAMP8 mice (control-R, *n* = 6; noise-R, *n* = 6), received donor fecal microbiota through transplantation. Briefly, the fecal pellets from control or HN donors were pooled in sterile saline (100 mg/ml), resuspended for 1 min, and then centrifuged at 1000*g* for 3 min. The supernatant was collected and delivered to the recipient mice via oral gavage (100 μl each recipient) within 10 min. The recipient mice were subjected to the microbiota transplant twice per week for 30 days.

### Statistics

Data presented in graphs indicate the group mean ± standard deviation and were analyzed using SPSS software (v.19.0; SPSS, Inc., Chicago, IL, USA). One-way analysis of variance with a post hoc least significant difference test was used to determine statistically significant differences between more than two groups. Data obtained from the 16S rRNA gene analysis were assessed using Wilcoxon rank sum tests. Redundancy analysis was performed using CANOCO 4.5. Spearman correlation coefficients (*R*, v3.1.2) were employed to measure the correlation between the differentially abundant genera and the metadata of cognitive performance and levels of ET, GABA, and 5-HT in mice. A *p* value < 0.05 was the threshold for statistical significance.

## Results

### Chronic noise exposure induces AD-like cognitive and pathological alterations in SAMP8 mice

**Fig. 1:**
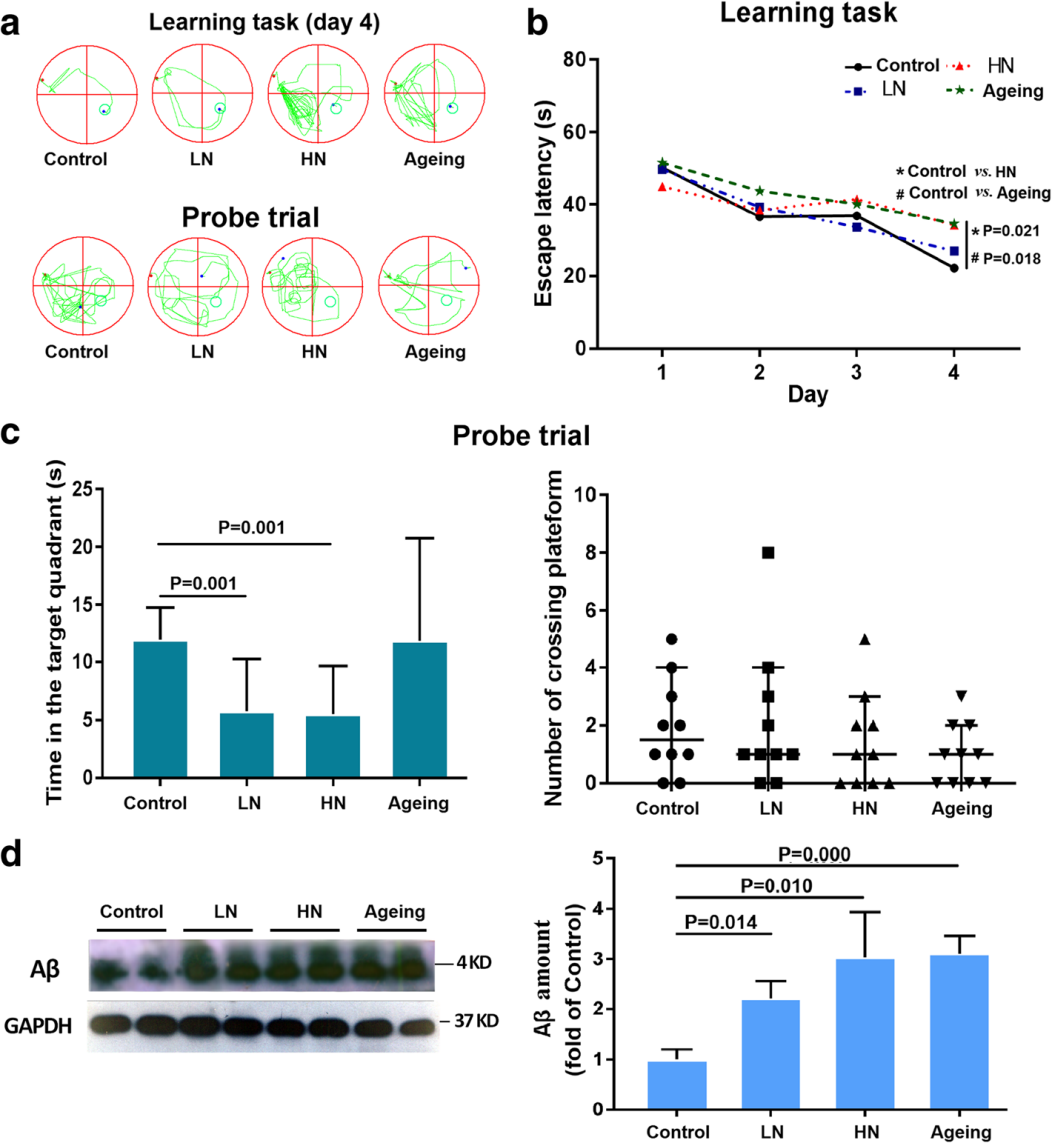
Chronic noise-induced Alzheimer’s disease-like cognitive and pathological alterations in SAMP8 mice. **a** Representative traces of SAMP8 mouse paths during the training phase and probe trial. **b** Effect of noise exposure on escape latency in the training phase (*n* = 10). **c** Effect of noise exposure on performance in the probe trial (*n* = 10). **d** Western blot analysis (left) and quantification (right) of Aβ in the hippocampus in each group. GAPDH was used as a loading control. Data are presented as the percent change relative to the control samples (*n* = 6). Data are shown as the mean ± standard deviation. HN high-intensity noise exposure, LN low-intensity noise exposure

Mice in all groups showed improvements in Morris water maze performance as indicated by decreasing escape latencies across the four training days. There were statistically significant differences in escape latency between the control group and the HN group as well as the aging group on day 4 (Fig. 1a, b). During the probe trial, the noise-exposed groups spent markedly less time in the target quadrant than the control group, although there were no statistically significant between-group differences in the number of platform location crossings despite a trend towards fewer crossings in the noise-exposed and aging groups (Fig. 1a, c).

To further evaluate the effects of chronic noise exposure on AD-like pathology, we determined the relative levels of Aβ in hippocampal tissues. Aβ amount was significantly increased in the LN and HN groups compared with the control group, with a higher level of significance in the HN group. Aβ was also significantly higher in the aging group than in the control group and similar to the level observed in the LN group (Fig. 1d). These data suggested that chronic noise exposure accelerated senescence-related cognitive and pathological alterations in a dose-dependent manner.

### Chronic noise exposure alters the gut microbiota in SAMP8 mice

The effects of chronic noise exposure on gut microbiota were determined by a 16S rRNA amplicon sequencing (16S sequencing)-based analysis of gDNA extracted from cecal content. A comparison of alpha diversity indices based on random subsampling of the reads of each sample did not reveal any significant between-group differences (Additional file 1: Figure S1A-C). At the phylum level, *Firmicutes*, *Bacteroidetes*, *Proteobacteria*, *Tenericutes*, *Chlorobi*, *Chloroflexi*, *Actinobacteria*, *Planctomycetes*, *Cyanobacteria*, and *Deferribacteres* were the dominant bacterial taxa (Additional file 1: Figure S1D). Among these phyla, *Firmicutes*, *Bacteroidetes*, and *Proteobacteria* were detected in all samples. At the genus level, *Lachnospiraceae*, *Bacteroides*, *Alistipes*, *Helicobacter*, *Odoribacter*, *Oscillibacter*, *Lachnoclostridium*, *Ruminiclostridium*, and *Prevotella* were highly abundant (Additional file 1: Figure S1E).

**Fig. 2:**
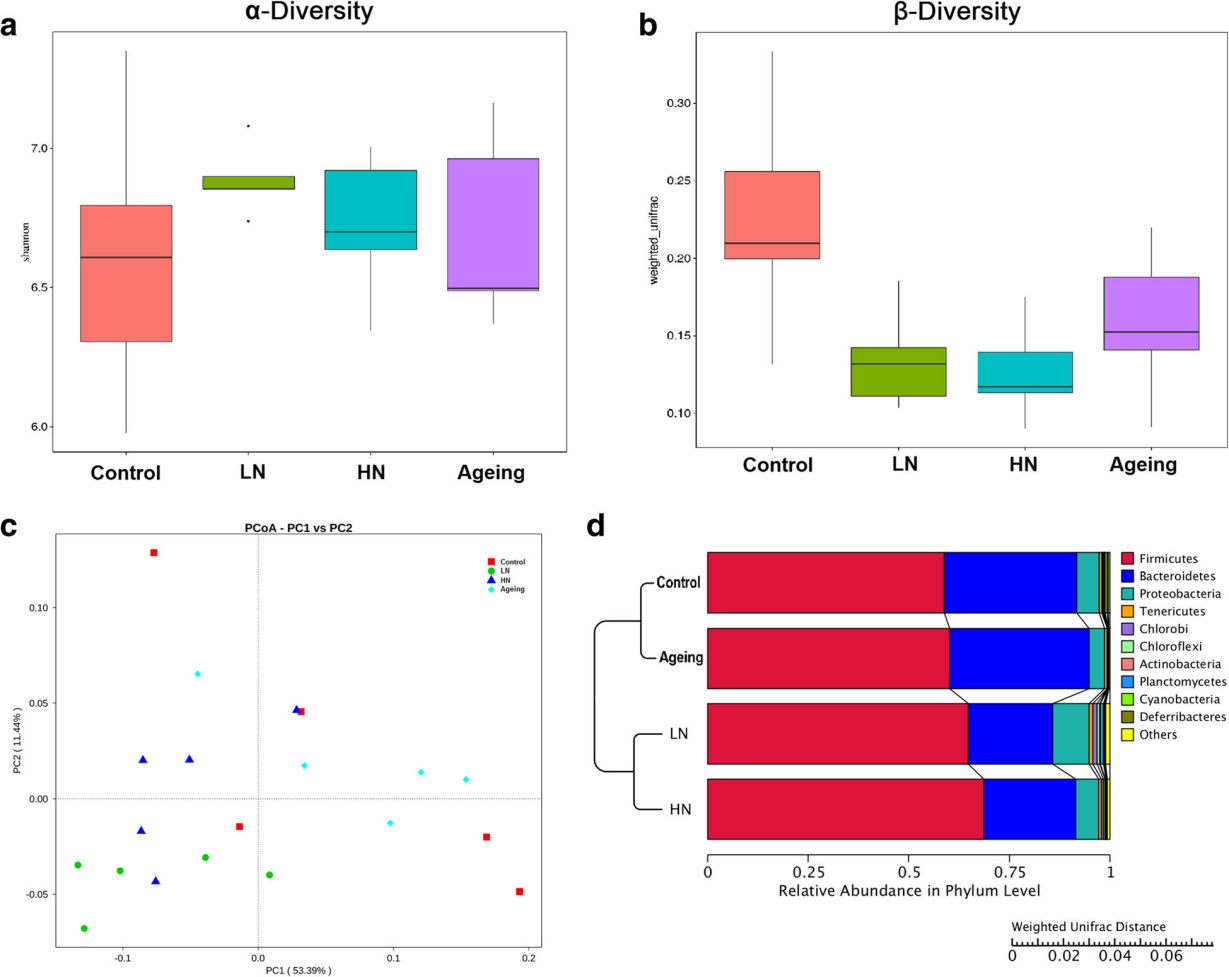
Summary of the gut microbial communities in each group. **a** Alpha diversity of the microbial community in each group analyzed by the Shannon diversity index (*n* = 5). **b** Intragroup β-diversity of the microbial community in each group measured by weighted UniFrac distance (*n* = 5). **c** Plots of weighted UniFrac-based PCoA in each group. **d** Relative abundances of predominant bacteria at the phylum level in each group (*n* = 5). HN high-intensity noise exposure, LN low-intensity noise exposure

There were no significant differences in alpha diversity of the microbial community between noise-exposed or aging mice and control mice (Fig. 2a); however, the microbiota community in the noise-exposed and aging SAMP8 mice exhibited lower intragroup beta diversity than that in control mice, with HN mice exhibiting the lowest level of diversity (Fig. 2b). Weighted UniFrac-based principal coordinate analysis (PCoA) also revealed distinct clusters of microbiota composition in the noise-exposed groups (Fig. 2c). Notably, taxonomic profiling demonstrated that the microbiota pattern of SAMP8 mice was altered by chronic noise exposure (Fig. 2d). These results suggested that chronic noise exposure facilitates adverse age-related changes in gut microbiota development.

**Fig. 3:**
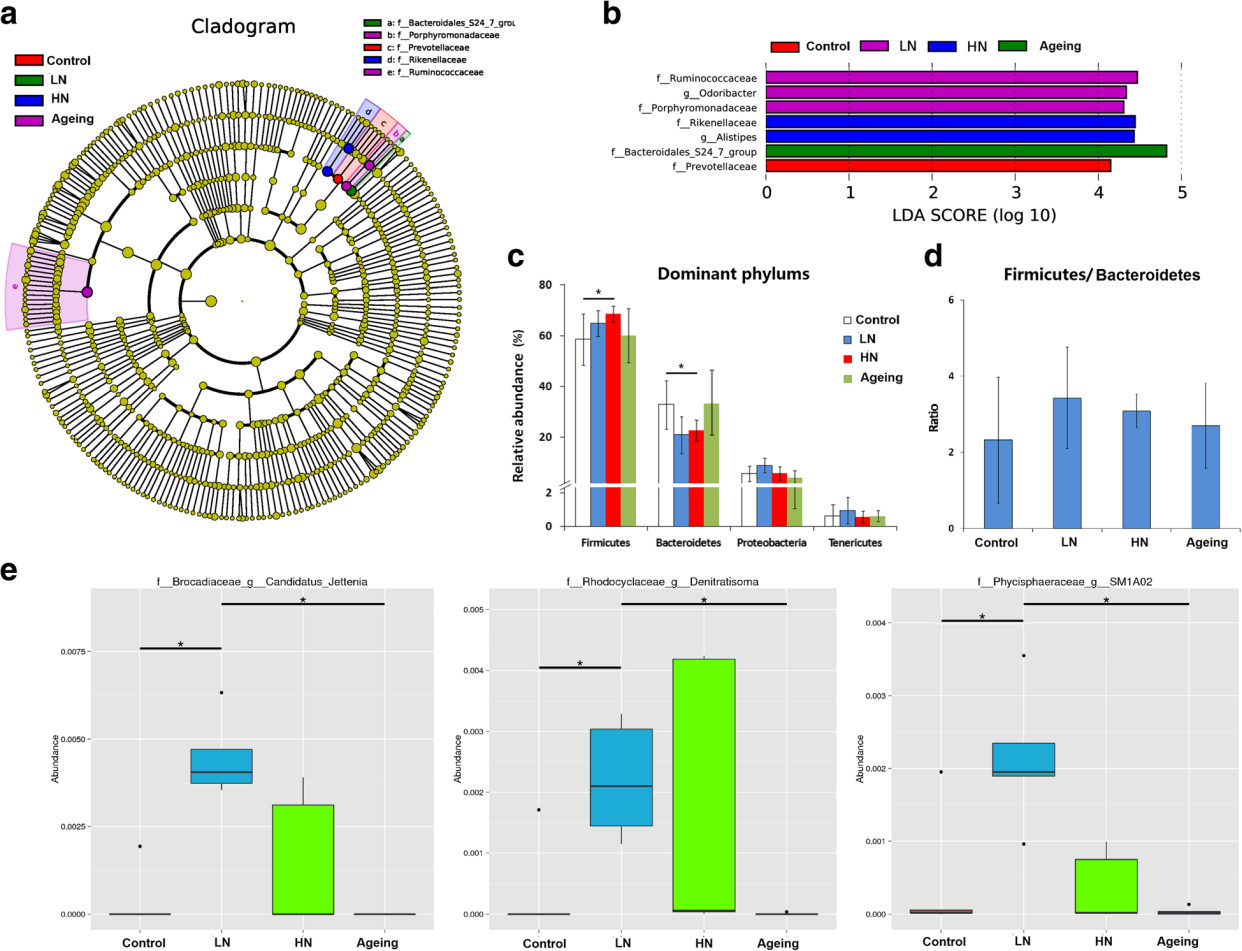
Characteristics of gut microbiota composition after chronic noise exposure. **a** The enriched taxa in the cecal microbiota of mice are represented in cladograms. The central point represents the root of the tree (bacteria), and each ring represents the next lower taxonomic level (phylum to genus). The diameter of each circle represents the relative abundance of the taxon (*n* = 5). **b** The most differentially abundant taxa in each group identified by LDA scores generated from the LEfSe analysis (*n* = 5). **c** Comparison of the relative abundances of the dominant phyla in all groups. **d** Ratio of *Firmicutes* to *Bacteroidetes* in each group. **e** Comparison of relative abundances at the bacterial genus level in all groups. *p < 0.05, Mann-Whitney *U* test. HN high-intensity noise exposure, LN low-intensity noise exposure

Linear discriminant analysis effect size (LEfSe) testing was used to further assess alterations in microbiota composition after 30 days of noise exposure. The structure and predominant bacteria of microbiota in each group are represented as a cladogram (Fig. 3a). The greatest difference in taxa from the phylum to the genus level was identified via the linear discriminant analysis (LDA) score (Fig. 3b). Most specific taxa were from two dominant phyla: *Firmicutes* and *Bacteroidetes*. At the phylum level, the abundance of *Firmicutes* was increased and that of *Bacteroidetes* was diminished by noise exposure (Fig. 3c). Both noise exposure and aging were associated with an increased ratio of *Firmicutes* to *Bacteroidetes* (Fig. 3d), which may have been associated with increased inflammation [20]. At the genus level, noise exposure in SAMP8 mice significantly increased the levels of *Candidatus Jettenia*, *Denitratisoma*, and *SM1A02* (Fig. 3e).

### Chronic noise exposure impairs intestinal and brain endothelial tight junctions in SAMP8 mice

**Fig. 4:**
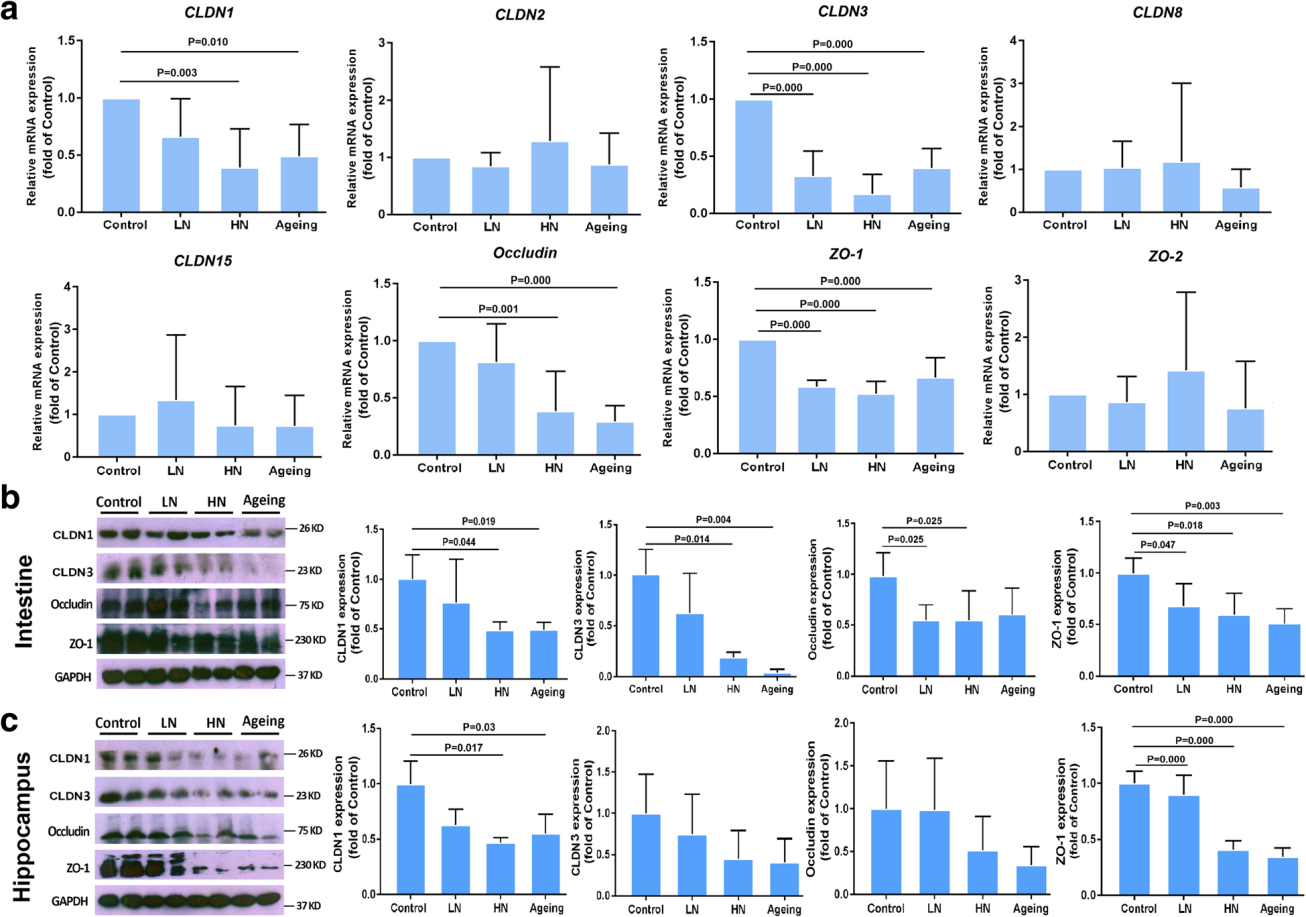
Chronic noise exposure and aging diminish intestinal and brain endothelial tight junction protein expression in SAMP8 mice. **a** mRNA expression levels of the tight junction components in SAMP8 mouse intestine samples (*n* = 6). **b** Protein expression levels of the main tight junction components in SAMP8 mouse intestine samples (*n* = 4). **c** Protein expression levels of the main tight junction proteins in the hippocampus of SAMP8 mice (*n* = 4). GAPDH was used as a loading control. Data are shown as the mean ± standard deviation. HN high-intensity noise exposure, LN low-intensity noise exposure

To evaluate the state of intestinal and blood-brain barrier (BBB) permeability after chronic noise exposure, we screened the gene and protein expression of the main tight junction using RT-PCR and Western blotting, respectively. Noise-exposed groups exhibited significantly lower mRNA expression of *CLDN1*, *CLDN3*, *occludin*, and *ZO-1* in the intestine than control mice, with a higher level of significance in the HN group. mRNA expression of *CLDN1*, *CLDN3*, *occludin*, and *ZO-1* was also markedly lower in the intestine of aging mice than in control mice and was similar to that of noise-exposed mice (Fig. 4a). There were no between-group differences in the mRNA expression of *CLDN2*, *CLDN8*, *CLDN15*, or *ZO-2*. Immunoblotting confirmed decreased intestinal expression of CLDN1, CLDN3, occludin, and ZO-1 in both noise-exposed and aging mice compared with control mice (Fig. 4b). Similar expression patterns of CLDN1 and ZO-1 were also observed in the hippocampus of noise-exposed groups and aging mice, and the expression of CLDN3 and occludin also showed downward trends in noise-exposed and aging mice, though the differences were not statistically significant (Fig. 4c). These results indicated that chronic noise exposure induced aging-like impairment in the epithelial integrity of the intestine and BBB in a dose-dependent manner.

### Chronic noise exposure causes blood neurotransmitter abnormalities and systemic inflammation in SAMP8 mice

**Fig. 5:**
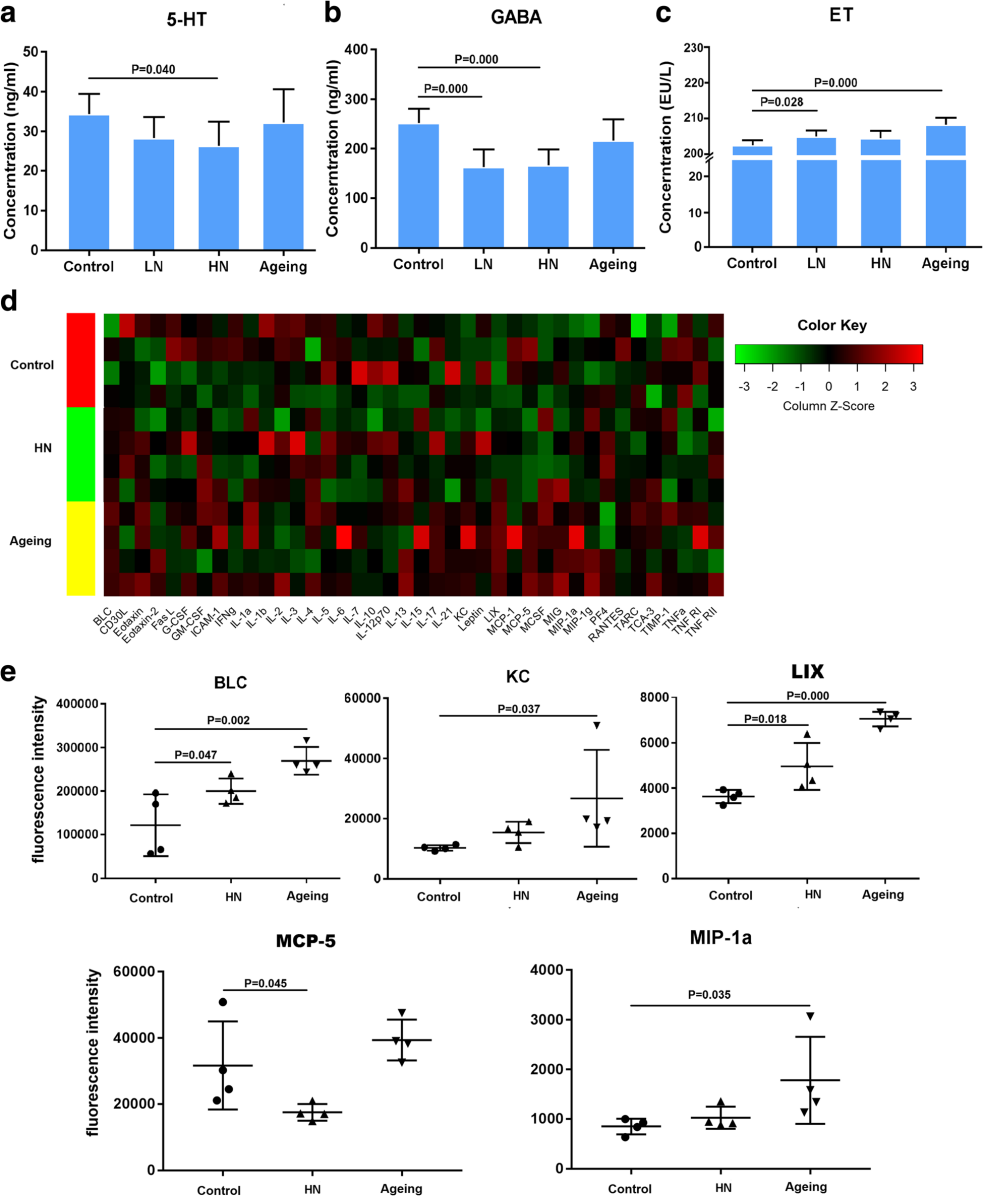
Chronic noise exposure produces abnormalities in serum neurotransmitters and chemokines in SAMP8 mice. **a**–**c** Enzyme-linked immunosorbent assay analysis of GABA, 5-HT, and endotoxin (ET) concentrations for each group (*n* = 6). **d** Heatmap showing the expression values of 40 serum cytokines measured by protein array (*n* = 4). **e** Normalized net intensities of cytokines showing significant intergroup changes in the protein array (*n* = 4). Data are shown as the mean ± standard deviation. HN high-intensity noise exposure, LN low-intensity noise exposure

To further explore the mechanisms underlying noise-induced changes in the microbiome-gut-brain axis, we measured the levels of GABA, 5-HT, and ET in the blood using ELISA. Noise exposure dose-dependently decreased 5-HT and GABA concentrations in the blood, whereas those in aging mice were not significantly different from the control values (Fig. 5a, b). ET was increased in the LN group and in aging mice compared with control mice (Fig. 5c). Taken together, these data suggest that chronic noise exposure may influence gut microbiota-related neurochemical and metabolic dysregulation, potentially contributing to the acceleration of aging and AD-related pathology.

Finally, we examined serum alterations in cytokines to investigate the effect of chronic noise exposure on systemic inflammation. A schematic of the chip format and images of control, noise-exposed, and aging group arrays in the Cy3 channel are shown in Additional file 2: Figure S2A and B. As shown in Fig. 5d and e, marked alterations were observed for a substantial number of pro-inflammatory cytokines; BLC and LIX were abundantly expressed in both the noise-exposed and aging groups compared with the control group, whereas KC and MIP-1a were only increased in aging mice, and MCP-5 was only reduced in noise-exposed mice. Alternatively, several major anti-inflammatory cytokines, including IL-4, IL-10, and IL-13, did not show significant alterations. Our findings indicate that noise exposure and aging may differentially influence the local cytokine environment in SAMP8 mice.

### Relationships among noise exposure, aging, and the microbiome-gut-brain axis

**Fig. 6:**
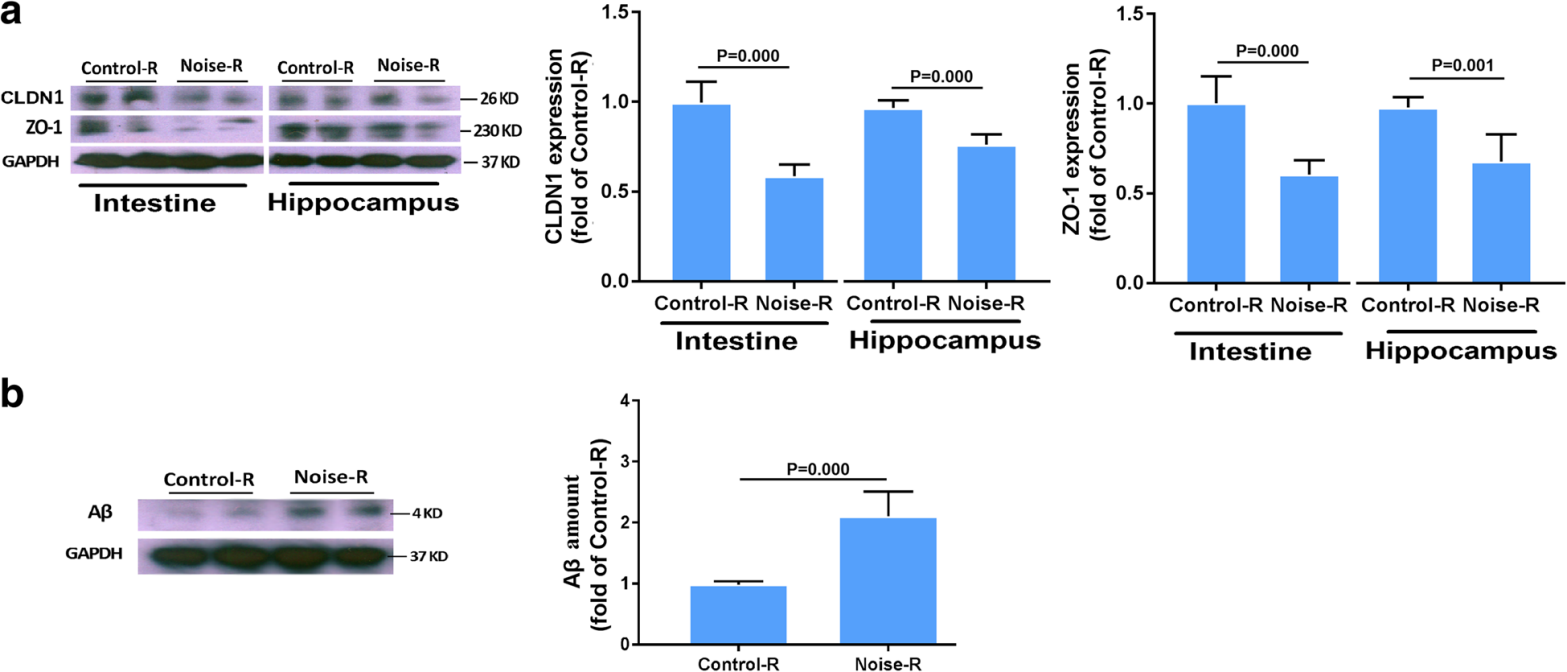
Effect of microbiota transplant on epithelial integrity and Aβ accumulation. **a** Western blot analysis (left) and quantification (right) of the main tight junction components in the intestine and hippocampus of control- and HN-microbiota recipient SAMP8 mice (*n* = 6). **b** Western blot analysis (left) and quantification (right) of Aβ in the hippocampus of control- and HN-microbiota recipient SAMP8 mice (*n* = 6). GAPDH was used as a loading control. Data are shown as the mean ± standard deviation. HN high-intensity noise exposure, LN low-intensity noise exposure

To determine the causality of altered microbiota, we transferred fecal microbiota from control or HN mice to age-matched male recipient mice (not exposed to noise). Immunoblotting confirmed the decreased expression of CLDN1 and ZO-1 in both the intestine and hippocampus of the HN-microbiota recipient mice compared to those in the control-recipient mice (Fig. 6a). The amount of Aβ in the hippocampus of the HN-microbiota recipient mice was increased compared to that in the control-recipient mice (Fig. 6b). These results indicate that host impairment in the epithelial integrity and AD-like changes are driven by the noise exposure-altered microbiota.

**Fig. 7:**
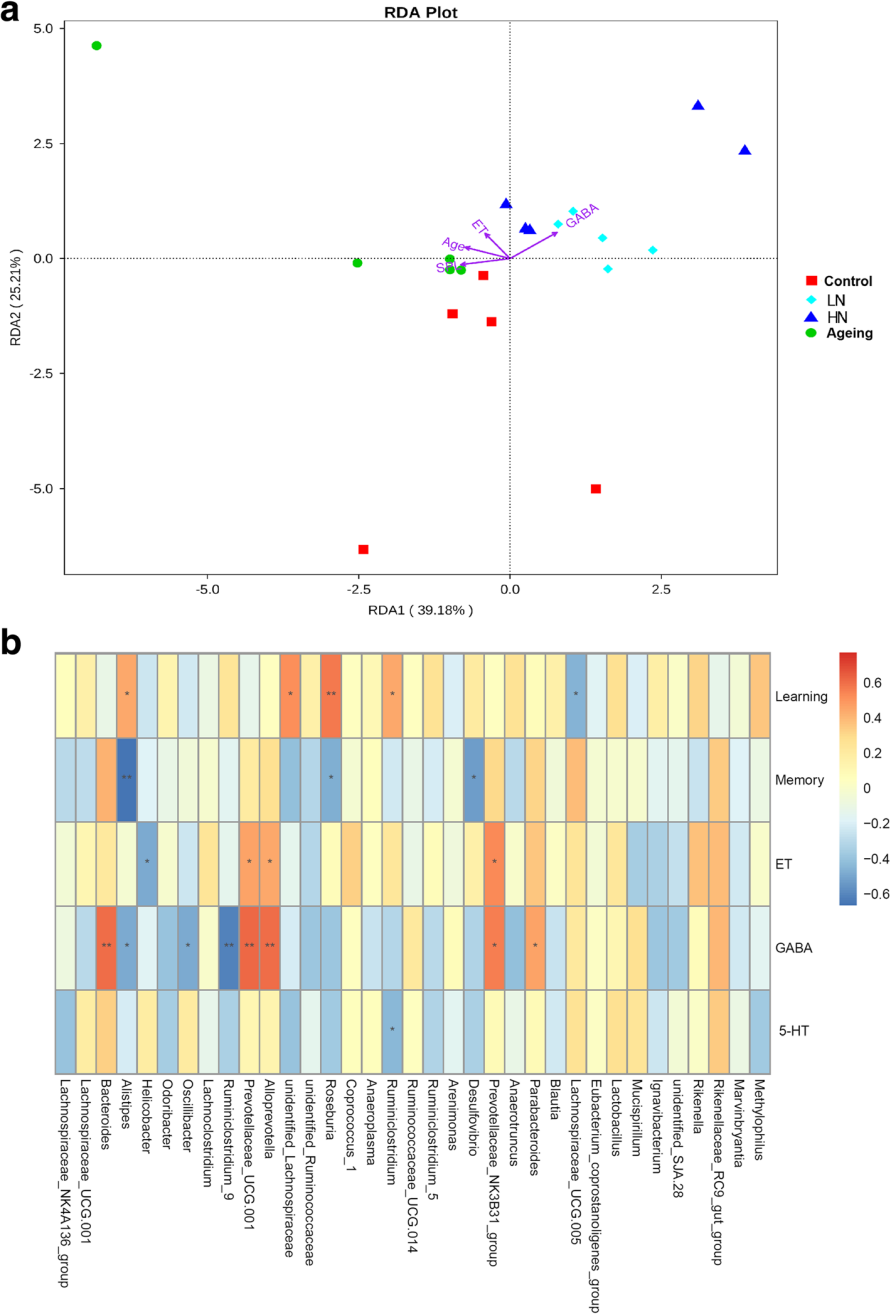
Relationships among noise exposure, aging, and the microbiome-gut-brain axis. **a** Redundancy analysis of the relationships among the main bacterial genera, noise intensity (sound pressure level, SPL), age, and levels of endotoxin (ET) and GABA. **b** Bacterial taxa in genera correlated with cognitive performance and levels of ET, GABA, or 5-HT in SAMP8 mice. The heatmap shows correlation coefficients generated from a Spearman correlation analysis. *p < 0.05 and **p < 0.01. HN high-intensity noise exposure, LN low-intensity noise exposure

Moreover, we investigated the correlations among altered OTUs, environmental factors, neurotransmitter levels, and cognitive abilities in all mice using a redundancy analysis and Spearman correlation analyses. The redundancy analysis revealed that the explanatory variables accounted for 64.4% of the total variation. Noise exposure and aging were the main positive environmental contributors to microbiota composition in mice, but they were negatively correlated with the GABA level (Fig. 7a). Spearman correlation analyses showed that, at the genus level, there were 5 OTUs correlated with escape latency during the training period in Morris water maze testing, including one negatively correlated genus (*Lachnospiraceae_UCG.005*) and four positively correlated genera (*Roseburia* and *unidentified-Lanchnospiraceae*). Three genera were negatively correlated with time spent in the target quadrant during the probe trial (memory performance); of these, *Roseburia* and *Alistipes* were significantly correlated with both types of cognitive performance tested in the Morris water maze. Four OTUs showed correlative relationships with the ET level in the blood, including one negative correlation (*Helicobacter*) and three positive correlations (including *Alloprevotella* and *Prevotellaceae_UCG.001*). Eight OTUs showed correlative relationships with the GABA level in the blood, including three negative correlations (including *Ruminiclostridium_9* and *Oscillibacter*) and five positive correlations (including *Bacteroides*, *Alloprevotella*, and *Prevotellaceae_UCG.001*). One OTU (*Ruminiclostridium*) was negatively correlated with the 5-HT level in the blood (Fig. 7b). These data suggested that the dysregulation of intestinal microbiota composition induced by noise exposure and aging might be linked to cognitive and neurochemical impairments in the SAMP8 mouse model of AD.

## Discussion

Previous studies have reported that chronic noise exposure, a common environmental health hazard, increases the risk of developing metabolic disorders [19, 21–24] and neurodegeneration [5–8]. In a previous study, we found that chronic noise exposure induced alterations in the gut microbiota accompanied by host immune responses and persistent abnormalities in glucose regulation [19]. Here, we expanded on these findings and found that chronic noise exposure was associated with a series of microbiome-gut-brain axis events, including changes in gut microbial community composition, disrupted epithelial barrier function in the intestine and BBB, and inflammatory changes associated with cognitive impairment. Thus, chronic noise may accelerate the appearance of certain biological markers of brain aging to facilitate the onset or progression of AD.

The principal risk factor for AD is aging. Aging represents a period of high vulnerability to unstable or adverse environmental conditions that accelerate cognitive impairment and hippocampal dysfunction [25, 26]. Numerous studies have been published on the association between noise exposure and aging and their causal roles in the development of AD-like pathological hallmarks such as massive neuronal and synapse loss as well as the accumulation of neuritic plaques (Aβ) and neurofibrillary tangles in the hippocampus and cortex [6–8, 27]. Our present results indicate that chronic noise exposure accelerated Aβ synthesis and cognitive decline in SAMP8 mice, consistent with previous studies [8, 17]. Furthermore, our study demonstrates that 30 days of noise exposure was sufficient to facilitate AD-like abnormalities in 3-month-old SAMP8 mice in a manner that mimicked abnormalities in older (8-month-old) SAMP8 mice.

Recent research suggests that the environment is a stronger contributor than genetics to gut microbiota composition [28]. Noise-exposed mice exhibited reductions in β-diversity and compositional changes in microbial communities that were more significant than those observed in the aging group, suggesting that microbiota alteration is a susceptible event that drives later gut-brain axis effects. The key microbiota characteristics in noise-exposed SAMP8 mice included reduced microbiota abundance and taxonomic diversity, which are considered hallmarks of aging. Chronic noise exposure also increased the ratio of *Firmicutes* to *Bacteroidetes* bacteria, an alteration that has been previously associated with increased ET levels and inflammation [20]; we also observed increased ET and inflammatory alterations after chronic noise exposure in the present study. Age-related inflammation is a chronic progressive pro-inflammatory response [29] that gradually disrupts the balance of the gut microbiota [30, 31], resulting in altered microbiota diversity and inflammatory homeostasis [32, 33]. The gut microbiota composition of elderly individuals is also affected by living environment [33]. Consistently, we found that the chronic noise exposure was positively correlated with aging-related alterations in microbiota composition. Finally, compositional differences in the microbiota of noise-exposed animals at the bacterial genus level also appeared to contribute to subsequent gut-brain axis disruption, although the underlying mechanisms of this alteration require further study.

The gut microbiota plays an important role in the synthesis of GABA and 5-HT [34, 35], which are major CNS neurotransmitters that regulate cognitive function. The inhibition of GABA and 5-HT biosynthesis by disturbance of the gut microbiota may affect pathological processes in AD [35–37]. Our observation of reduced blood GABA and 5-HT levels in noise-exposed mice in close correspondence to gut microbiota disruption and increased brain Aβ production provides further evidence for the effects of chronic noise on the microbiome-gut-brain axis.

Tight junctions consist of transmembrane proteins (claudins and occludins) and cytoplasmic membrane proteins (ZO-1 and ZO-2) and play a major role in the functional maintenance of the BBB and intestinal epithelial barrier [38–41]. Regulation of tight junction proteins by the intestinal microbiota has been reported for the intestinal epithelial barrier [40] and the blood-testis barrier [42]. Our data revealed that both aging and chronic noise exposure dysregulated the mRNA and protein expression of tight junction proteins, including claudin 1, claudin 3, occludin, and ZO-1 in the intestine and brain, demonstrating a widespread defect in intestinal barrier and BBB integrity. Additionally, these observations suggest that endothelial tight junction protein expression in the BBB and intestine is sensitive to changes in the intestinal gut microbiota and may mediate the progression from systemic inflammation and neurochemical dysregulation to neurodegeneration.

Gut permeability is commonly associated with an altered immune response [43]. Alterations in gut microbiota can lead to increased intestinal permeability associated with endotoxin absorption and consequently endotoxemia [44]. Thus, levels of endotoxin in the blood can represent leaky gut in addition to inflammation. Studies have indicated that ET may also play a role in hippocampal Aβ accumulation, cognitive deficits, and AD progression [45, 46]. In a previous study, we demonstrated a chronic noise-induced intestinal inflammatory response [19] and reciprocal activation of pro-inflammatory cytokines and astrocytes, which could cause a positive feedback loop resulting in CNS abnormalities [8]. In the present research, we found that chronic noise exposure or aging increased systemic inflammation as evidenced by ET elevation and the upregulation of pro-inflammatory cytokines in the blood. Thus, increased inflammation combined with decreased barrier integrity at the level of the BBB may account for the effect of chronic noise exposure on hippocampal Aβ deposition and cognitive performance via the microbiome-gut-brain axis.

Finally, the present study showed that, at the genus level, gut *Roseburia* and *unidentified-Lanchnospiraceae* were positively correlated with spatial learning and memory performance in SAMP8 mice. Similarly, Bajaj et al. reported a positive association between *Roseburia* and cognitive performance [47]. *Alloprevotella* and *Prevotellaceae* were positively correlated with ET level, a key biomarker of the systemic inflammatory response; this may be because these bacteria are gram-negative and thus are major sources of ET. GABA, the major inhibitory neurotransmitter in the CNS, is synthesized by *Bacteroides* and *Prevotella* in the gut [48], and *Bacteroides* has been associated with depression and social deficits in humans [13, 49]. There are currently no other reports on the other genera correlated with cognition, ET, or neurotransmitter levels in the present study.

The major limitation of this study is that the differences in auditory sensitivity frequencies between mice and human beings were not fully considered. The commonly used laboratory mice have hearing with sensitivity frequencies in the audible and ultrasonic range (1500 to 80,000 Hz), which is far beyond the range of human hearing (20 to 20,000 Hz) [50]. In the present study, the main spectrum of the noise emitted from the speaker was in the range of 400 to 6300 Hz (measured using on third octave bands), which coincides with the sensitivity frequency of mice hearing. Additionally, noise exposure dose-dependently increased corticosterone concentrations in the blood (Additional file 3: Figure S3), which demonstrates that the chosen range of noise frequency in the present study had a confirmative effect on the mice.

## Conclusions

The study provides a systematic investigation of the effects of chronic noise exposure on the microbiome-gut-brain axis in the senescence-accelerated prone mouse. Our results indicate that chronic noise exposure altered the gut microbiota, accelerated age-related neurochemical and inflammatory dysregulation, and facilitated AD-like changes in the brain of SAMP8 mice (Table 3). These findings enhance our understanding of the etiological association between chronic noise exposure and dysregulation of the microbiome-gut-brain axis and suggest that modulation of the gut microbiota is a potential intervention for protecting against the detrimental effects of chronic noise exposure. We have reason to speculate that AD may begin with imbalance in the gut microbiota; the present study highlights the possibility that chronic noise and aging produce a synergistic effect on gut microbiota dysbiosis and gut-brain axis abnormalities, though this hypothesis requires further study.

**Table 3:** Summarized comparison of changes caused by noise exposure and aging

Feature	Compared to control	
	Noise-induced changes	Aging-dependent changes
Cognition		
Learning	Decreased	Decreased
Memory	Decreased	n.s.
Aβ amount	Increased	Increased
Gut microbiota		
Alpha diversity	Decreased (n.s.)	Decreased (n.s.)
Beta diversity	Decreased (n.s.)	Decreased (n.s.)
PCoA	Distinct	n.s.
Microbiota pattern	Distinct	n.s.
Firmicutes	Increased	n.s.
Bacteroidetes	Decreased	n.s.
Intestinal tight junctions		
CLDN1	Decreased	Decreased
CLDN3	Decreased	Decreased
ZO-1	Decreased	Decreased
Occludin	Decreased	Decreased (n.s.)
Brain tight junctions		
CLDN1	Decreased	Decreased
CLDN3	Decreased (n.s.)	Decreased (n.s.)
ZO-1	Decreased	Decreased
Occludin	Decreased (n.s.)	Decreased (n.s.)
Neurotransmitters		
GABA	Decreased	n.s.
5-TH	Decreased	n.s.
Inflammation		
ET	Increased	Increased
BLC	Increased	Increased
KC	n.s.	Increased
LIX	Increased	Increased
MCP-5	Decreased	n.s.
MIP-1a	n.s.	Increased

*n.s.* not significant

## Additional files

Additional file 1: **Figure S1.** Dominant bacteria taxa at the phylum (A) and genus (B) level from all samples. Only phyla with ≥ 0.1% abundance and genera with ≥ 0.1% abundance detected in ≥ 5 samples are shown. (TIF 334 kb)

Additional file 2: **Figure S2.** Protein array format. Representative images of the control (A), low intensity noise exposure (LN) (B), high intensity noise exposure (HN) (C), and aging (D) group cytokine arrays in the Cy3 channel. A key to the location of the spotted primary antibodies is shown in Table 2. (TIF 3812 kb)

Additional file 3: **Figure S3.** Chronic noise exposure results in abnormal serum corticosterone levels in SAMP8 mice. Enzyme-linked immunosorbent assay analysis of corticosterone concentrations for each group (*n* = 8). Data are shown as the mean ± standard deviation. HN, high intensity noise exposure; LN, low intensity noise exposure. (TIF 130 kb)

## Abbreviations

5-HT: 5-Hydroxytryptamine
Aβ: Amyloid-β
AD: Alzheimer’s disease
ApoE: Apolipoprotein E
BBB: Blood-brain barrier
BLC: B lymphocyte colony
CLDN: Claudin
ELISA: Enzyme-linked immunosorbent assay
ET: Endotoxin
GABA: Gamma-aminobutyric acid
IL: Interleukin
KC: Platelet-derived growth factor-inducible protein KC
MIP-1a: Macrophage inflammatory protein 1-alpha
LEfSe: Linear discriminant analysis effect size
LIX: LPS-induced CXC chemokine
MCP-5: Monocyte chemoattractant protein-5
OTU: Operational taxonomic units
SAMP8: Senescence-accelerated mouse prone 8
SPL: Sound pressure level
ZO-1: Tight junction protein 1
