# Supplementary figures and images for: Effects of chronic noise exposure on the microbiome-gut-brain axis in senescence-accelerated prone mice: implications for Alzheimer’s disease
Source: J Neuroinflammation. 2018 Jun 22;15:190. doi: 10.1186/s12974-018-1223-4 (PMC6015475; doi:10.1186/s12974-018-1223-4)

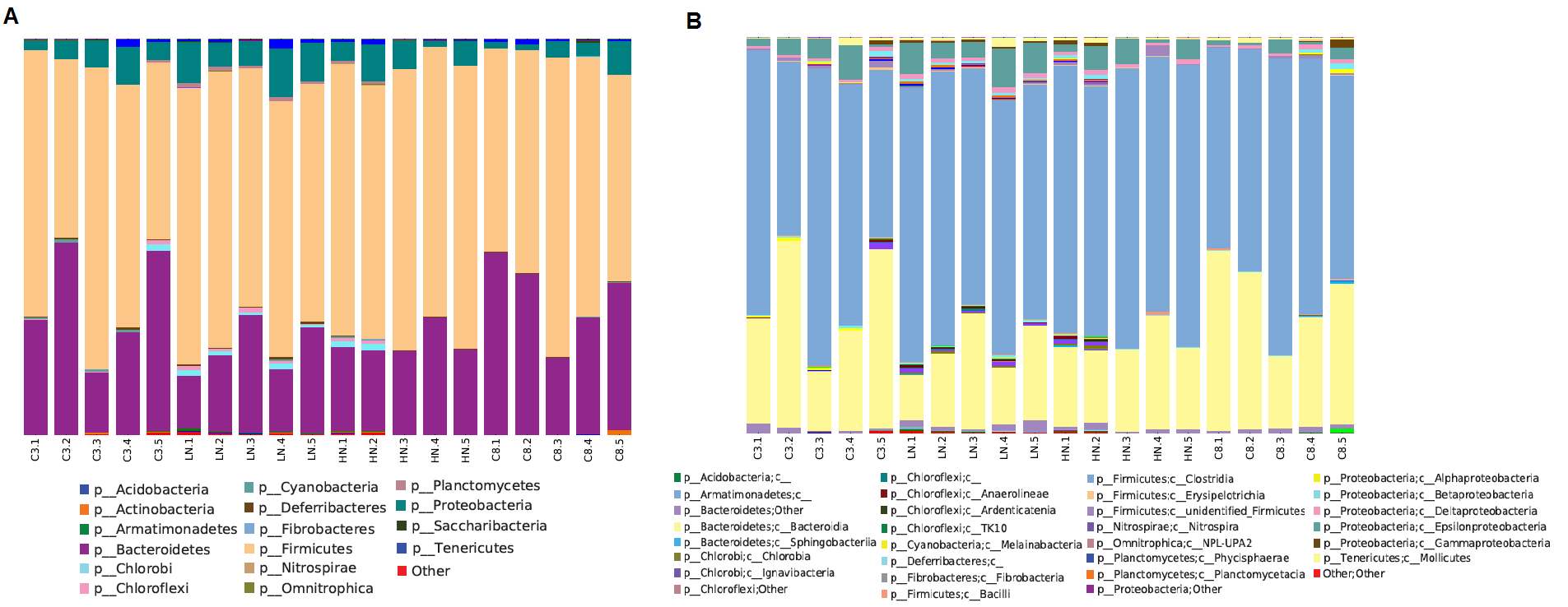

Supplement: Supplementary file 1 — Figure S1. Dominant bacteria taxa at the phylum (A) and genus (B) level from all samples. Only phyla with ≥ 0.1% abundance and genera with ≥ 0.1% abundance detected in ≥ 5 samples are shown. (TIF 334 kb) [file 12974_2018_1223_MOESM1_ESM.tif]

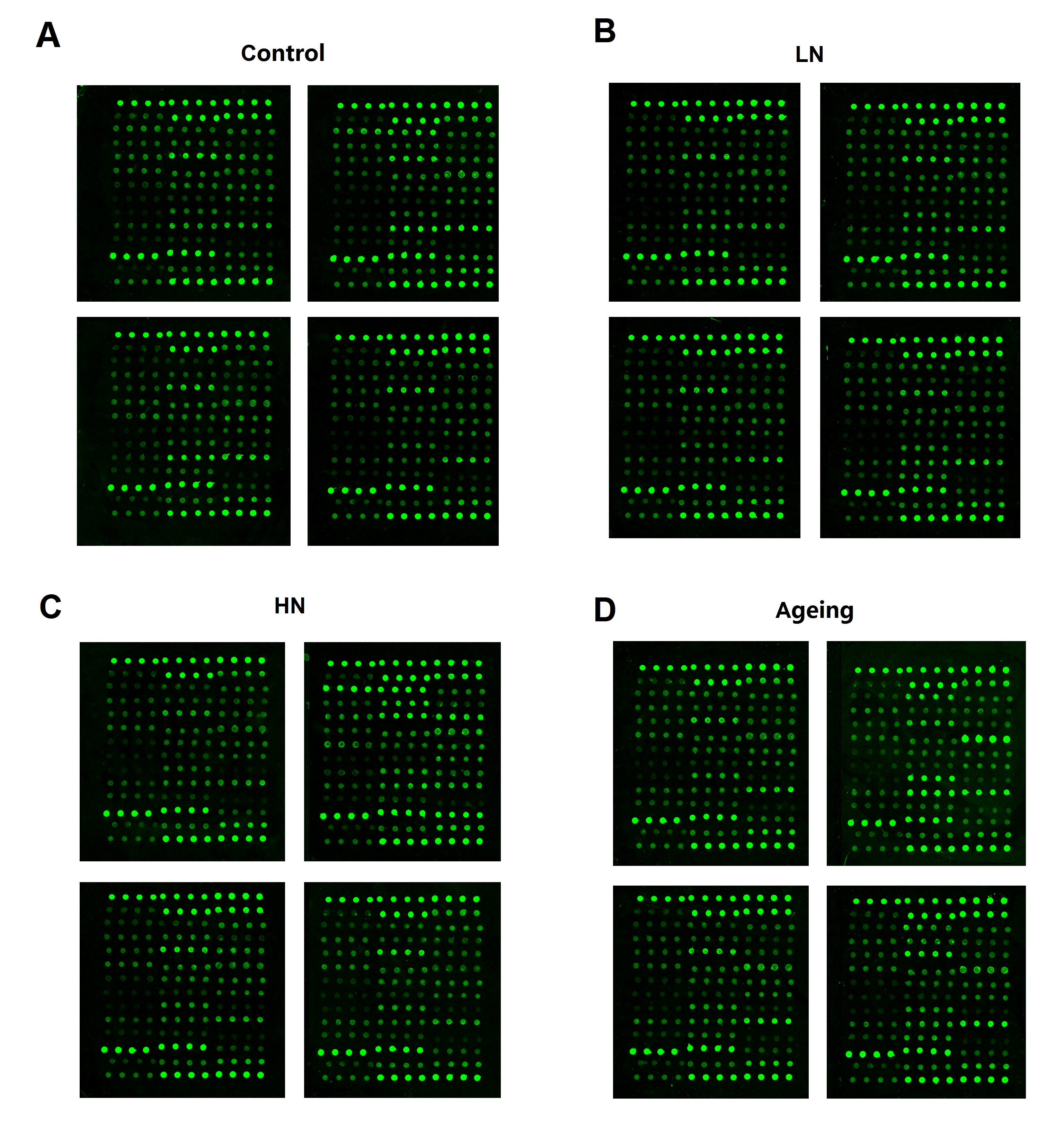

Supplement: Supplementary file 2 — Figure S2. Protein array format. Representative images of the control (A), low intensity noise exposure (LN) (B), high intensity noise exposure (HN) (C), and aging (D) group cytokine arrays in the Cy3 channel. A key to the location of the spotted primary antibodies is shown in Table 2. (TIF 3812 kb) [file 12974_2018_1223_MOESM2_ESM.tif]

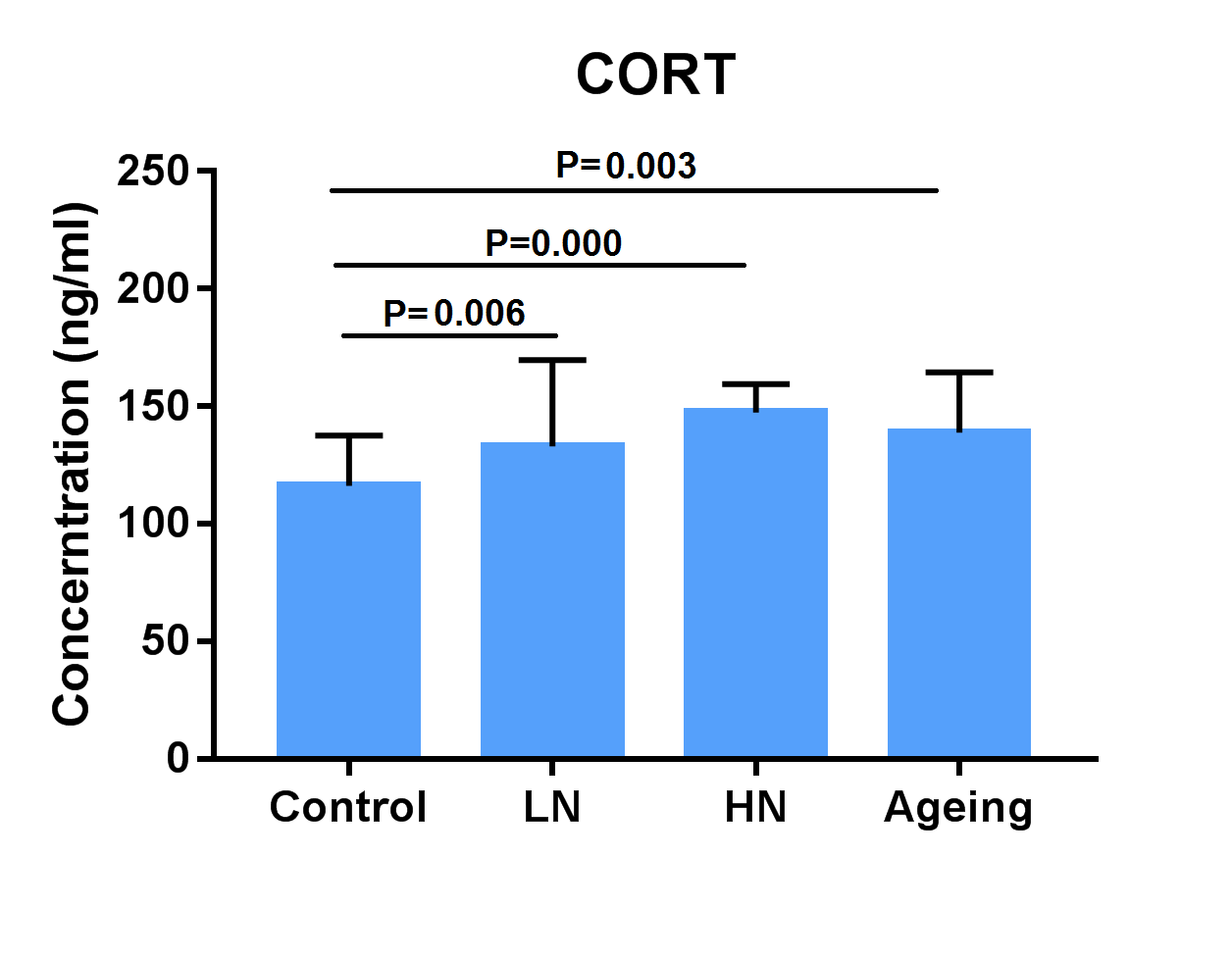

Supplement: Supplementary file 3 — Figure S3. Chronic noise exposure results in abnormal serum corticosterone levels in SAMP8 mice. Enzyme-linked immunosorbent assay analysis of corticosterone concentrations for each group (n = 8). Data are shown as the mean ± standard deviation. HN, high intensity noise exposure; LN, low intensity noise exposure. (TIF 130 kb) [file 12974_2018_1223_MOESM3_ESM.tif]
